# Annotations

**Published:** 1900-10-06

**Authors:** 


### Annotations.

### A Curious Vacation Course.

During the vacation one of the rooms at University College has, we understand, been occupied by a class of undertakers anxious to acquire familiarity with the art of embalming. It may, we think, be properly laid down as a general principle, that when death has taken place, the sooner a man's body is resolved into its primal elements the better, and that all attempts to prevent its dissolution are to be deprecated. Where it is necessary for legal purposes—as, for example, in case of disputed identity—to preserve a body, then the process of embalming finds its proper place. We cannot, however, but feel that so far as modern "embalming" forms part of the more or less ridiculous customs as to lying in state and dressing up the body, which seem of late to have taken root in America—a mere survival and rehabilitation of the "wakes" of old—the whole proceeding is one to be discouraged.

### Handwriting and Health.

We have received a pamphlet entitled *Why Should the Teaching of Sloping Handwriting be made an Offence Punishable at Law?* The reason stated for this rather alarming demand is that the attitude of the body necessary for the production of sloping writing tends to spinal curvature in the writer. The author of the pamphlet, Mr. John Jackson, brings forward statements from many medical men to prove his contention. Mr. Bernard Roth, in a volume on *Lateral Curvature of the Spine*, says: "The position of writing as generally practised is, more frequently than anything else, an initial cause in most cases of lateral and other curvatures not due to diseased bone or infantile paralysis." Mr. Noble Smith, of the City Orthopædic Hospital, says that "the twisted and curved position of the spine caused by writing is doubtless a very potent factor in the production of lateral curvature. The more slanting the writing, the worse the position." Other medical men have added their testimony to the same effect, and some German doctors declare that about half the school children they have examined have been suffering from spinal curvature, which they trace to the teaching of sloping writing. That the state of affairs is equally bad in this country is unlikely, for sloping writing is not so common here, and is steadily going out of fashion. Still it is worth while to bring forward and impress upon the teachers the fact that many medical men are of opinion that the posture necessary for sloping writing is vicious and unwholesome. Mr. Noble Smith "emphatically advises that upright be universally substituted for slanting writing." Mr. Jackson traces myopia, as well as spinal disease, to the style of writing which he holds in such detestation, and records with satisfaction the report of a French Commission on Public Instruction which says: "The Commission thinks that great progress will be made by exacting vertical writing on straight paper, the body erect. Thus both spinal curvature and short sight will be avoided altogether." While we think that there is much that is reasonable in Mr. Jackson's contention, we do not think that it need be supported by such drastic measures as he desires. After all, schoolmasters are not unintelligent men, and can be persuaded to teach upright writing if it is shown to be better, without the threat of penal servitude.

### Competition in Fees.

Protests continue to be made concerning the low fees offered to medical examiners by certain insurance companies. Instances are given of the voluminous questions to be answered, of the searching examination required, and of the ridiculously small fees which are to be paid, and it seems to be generally recognised that the condition of affairs is very sad and very bad—all of which is true enough. But it is also admitted that should any examiner in London refuse to sign the "parsimonious document" which has given rise to the outcry he knows full well that "there would be a dozen medical men within a radius of half a mile, needy, struggling practitioners, who would come forward and accept the terms and fees offered." This is the root of the matter—the absolute lack of union among medical practitioners in regard to everything which concerns their pockets—and until this is altered nothing will be done. It is no use growling about injustice, or declaiming against "sweating." Doctors, like other folk, are open to the influence of individual and immediate self-interest, and the man who shall discover and make clear to his brethren some strong bond of union, founded upon the ordinary human motive of self-interest, which shall bind together those at the top and those at the bottom of the ladder, will do more for his profession than a whole army of preachers holding forth about the injustice and degradation of the present system. Such a bond of union, however, is not easy to find, for in medicine, as in other affairs, the man in the coach takes a different view from the man in the gutter.

ANNOTATIONS—*continued.*

### Glasgow's Opportunity.

The fact that plague still keeps dribbling on, week after week, in Glasgow, serious as it may appear to those who imagined that our sanitary organisation would be able at once to stamp out any such outbreak, is neither matter for surprise nor for fresh anxiety.

As we pointed out at the time, the manner in which the disease first showed itself in Glasgow—so different from that in which the few cases met with in London had been discovered—was such as to cause considerable apprehension among those who best knew the habits of plague; for a "focus" of disease had become established before it was found out. But the mere continuance of the disease, and the upspringing of fresh cases now and again, is nothing to be discouraged about; it would, indeed, have been very surprising if the authorities had been able by a stroke to put an end to an infection which had already enjoyed such opportunities for diffusion.

Every credit is due to the sanitary authorities, not only at Glasgow but in all our great ports, for the precautions they have taken and for the immense amount of work they have done during many months past in endeavouring to prevent the importation of plague. But the proof of the pudding is in the eating, and the lesson taught by the Glasgow affair is that dependence must not be placed upon even the most elaborate precautions, that the most carefully devised efforts will not keep plague out of a country, and that each sanitary authority must be prepared to fight the disease within its own borders as Glasgow has had to do. To enable us to do this with good effect, increased knowledge of the mode by which the disease is transmitted from man to man is of the first importance; and here comes Glasgow's opportunity, for it must be confessed that, much as we know about the clinical history of the disease and about the bacillus by which it is caused, our knowledge is far from complete in regard to the manner in which the infection passes from case to case.

The history of infectious diseases has again and again shown that the manner in which infection spreads is not best studied in the midst of great epidemics, nor in places where such diseases are most prevalent. Where opportunities for infection exist on every side it is an almost hopeless task to trace an epidemic through its various wanderings, or to discover the source of infection in any given case. But when the disease is an exotic, when the cases are so few and far between as to render it probable that any link discovered between one patient and another is the real link by which the infection has travelled, then the investigator has a chance, and may with some hope of success attempt to unravel the tangled skein.

For all the thousands of people who used to die of cholera in India every year, and notwithstanding the experience gained in other countries when a vast epidemic swept like a great wave of death around the world, the manner in which the infection was spread remained a mystery until a more or less isolated outbreak among a few people who habitually drew their water from a particular pump in Broad Street, enabled Dr. Snow, by eliminating other sources of infection, and by tracing all the cases to this one pump, to demonstrate the fact that cholera was a water-borne disease.

And now in regard to plague, Glasgow has its opportunity in the very fact that the outbreak which it now suffers from is a small one, and that the disease is an absolute exotic.

The rats, the fleas, the other parasites of man, the dust, the dirt, the overcrowding, the want of light and air, the scratches on the feet or fingers, all the various circumstances which have from time to time been put forward as the means by which infection is conveyed or predisposition to it is developed all want inquiring into, and it is much to be hoped that the complete investigation, which is sure, we imagine, to have been made as to every detail regarding each case in such an interesting little outbreak as that which has now for some weeks been in progress in Glasgow, will eventuate in some useful addition of definite knowledge to the not too copious store of that commodity which we already possess in regard to the manner in which plague spreads in epidemic form.

### Malaria: a Crucial Experiment.

All scientific men who have watched and have admired the careful work which has led up to the establishment of the mosquito theory of malaria must feel great satisfaction at the experimental proof which has lately been given of its correctness, and not only scientific men, but men of all nations and degrees must feel a debt of gratitude to Dr. Patrick Manson, whose investigations have done so much in leading up to our present knowledge on the subject; to Dr. Sambon and Dr. Low, who have exposed themselves to the malignant infection of the Campagna; and to Mr. Thurburn Manson (son of Dr. Patrick Manson), who by allowing himself to be bitten by infected mosquitos, and by suffering from the disease in consequence, has given a final proof that these insects are the means by which malaria is distributed.

The two experiments hang together.

In the one Dr. Bignami and Dr. Bastianelli, having obtained some mosquitos, allowed them to feed upon a patient suffering from malaria of the tertian type. This was done in Rome, and the mosquitos were then transmitted to the London School of Tropical Medicine, where they were allowed to feed upon the hand of Mr. Thurburn Manson. In due time fever developed, and not only was it obvious to those who watched the case that he was suffering from the same disease as the patient from whom the mosquitos had derived the infection, but examination of his blood showed the presence of the specific parasites by which this disease is caused. It is satisfactory to be told that under treatment by quinine the disease has been brought to an end, and that the patient is all right again. The other experiment is that by Dr. Sambon, Dr. Low, and Signor Terzi, which we described at the time when it was commenced. These gentlemen, with their two Italian servants, have since early in July—that is, through the most dangerous season of the year—been living in one of the worst parts of the Campagna, a place where the permanent inhabitants all suffer from malaria. They have been going about the country quite freely during the day, but from sunset to sunrise have kept within their hut, which was so constructed as to prevent the ingress of mosquitos. During all this time they have enjoyed perfect health, while their neighbours suffered from fever as usual. Thus these experiments have shown on the one hand that mosquitos which have been fed upon a malarial case are capable of infecting a healthy man in a far country, who has not been exposed to any other source of infection; and on the other hand that, in a country absolutely saturated with malaria, it is possible to escape the disease by the simple precaution of excluding the infection-carrying mosquitos during the hours at which they habitually feed. Nothing could be neater or more convincing. Of course we knew it all before; but to impress it on the public mind some such dramatic proof as this was wanted, and it is very satisfactory to find that the experiment "came off" so well.

